# Membrane Transporters for Amino Acids as Players of Cancer Metabolic Rewiring

**DOI:** 10.3390/cells9092028

**Published:** 2020-09-03

**Authors:** Mariafrancesca Scalise, Lara Console, Filomena Rovella, Michele Galluccio, Lorena Pochini, Cesare Indiveri

**Affiliations:** 1 Unit of Biochemistry and Molecular Biotechnology, Department DiBEST (Biologia, Ecologia, Scienze della Terra), University of Calabria, Via Bucci 4C, 87036 Arcavacata di Rende, Italy; mariafrancesca.scalise@unical.it (M.S.); lara.console@unical.it (L.C.); filomena.rovella@unical.it (F.R.); michele.galluccio@unical.it (M.G.); lorena.pochini@unical.it (L.P.); 2 CNR Institute of Biomembranes, Bioenergetics and Molecular Biotechnologies (IBIOM) via Amendola 122/O, 70126 Bari, Italy

**Keywords:** amino acids, tumors, transporters, metabolism

## Abstract

Cancer cells perform a metabolic rewiring to sustain an increased growth rate and compensate for the redox stress caused by augmented energy metabolism. The metabolic changes are not the same in all cancers. Some features, however, are considered hallmarks of this disease. As an example, all cancer cells rewire the amino acid metabolism for fulfilling both the energy demand and the changed signaling routes. In these altered conditions, some amino acids are more frequently used than others. In any case, the prerequisite for amino acid utilization is the presence of specific transporters in the cell membrane that can guarantee the absorption and the traffic of amino acids among tissues. Tumor cells preferentially use some of these transporters for satisfying their needs. The evidence for this phenomenon is the over-expression of selected transporters, associated with specific cancer types. The knowledge of the link between the over-expression and the metabolic rewiring is crucial for understanding the molecular mechanism of reprogramming in cancer cells. The continuous growth of information on structure–function relationships and the regulation of transporters will open novel perspectives in the fight against human cancers.

## 1. Introduction

It is well established that the metabolism of cancer cells is profoundly altered with respect to their normal counterparts. This phenomenon is currently known as metabolic rewiring. On the one hand, this phenomenon is responsible for sustaining the dramatically increased growth rate of cancer cells, on the other for compensating the redox stress caused by the increased energy demand [1,2,3,4].

Over time, it has become increasingly evident that the changes to the metabolism are not homogeneous among different cancer types and, within same cancer, among different stages of development. Furthermore, cancer cells can adapt to oxygen availability, surrounding environment, the supply of nutrients and last but not least, pharmacologic treatments. Notwithstanding the great variability, there are some features which are hallmarks of cancers due to their presence across different cancer types. One of the common traits is that cancer cells modify the metabolism of amino acids. These are either nutrients or scaffold molecules, and are thus very important for the increased growth conditions.

**Figure 1:**
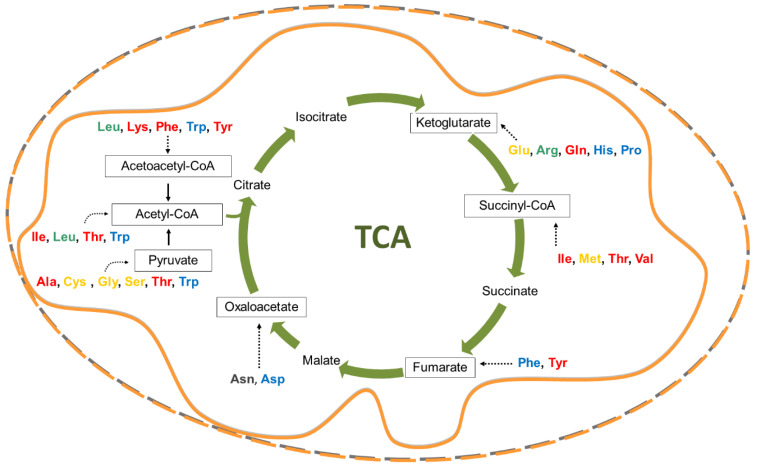
Schematic representation of amino acid catabolic processes. The Tricarboxylic acid cycles reactions are represented by green arrows conducting to the respective intermediate products. The boxed intermediates, including the non-TCA intermediate Acetoacetyl-CoA, are the sites of amino acid confluences for carbon skeleton oxidation. In red, amino acids mainly involved in energy production; in yellow, amino acids mainly involved in redox balance; in blue, amino acids mainly involved in cell invasion and metastasis; in grey, amino acids mainly involved in apoptosis; in green, amino acids mainly involved in mTOR activation. The continuous or dotted black arrows represent non-TCA reactions or pathways, respectively.

Indeed, in addition to their proteinogenic role, amino acids are employed in versatile ways for producing biomass, for signaling purposes, and for deriving energy [5,6,7]. For this last purpose, the cell pathway collecting all the amino acids, via anaplerotic reactions, is the TCA (tricarboxylic acid cycle) (Figure 1).

These premises fully justify the sizable number of articles dealing with amino acids utilization and cancer, published in the last decade. A prerequisite for increasing the utilization of most of the amino acids is the over-expression of the membrane transporters responsible for their absorption from the surrounding environment [8,9,10,11]. Among the top amino acids linked with cancers, there are glutamine, glutamate, leucine, aspartate, asparagine, methionine, and serine [1,2,3,12].

**Figure 2:**
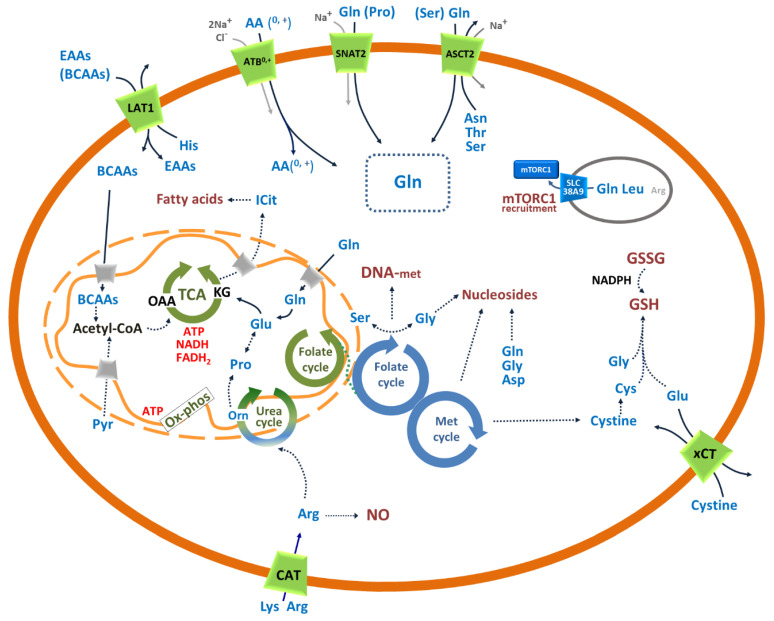
Schematic representation of specific pathways involved in changes of amino acid transport and metabolism occurring in cancer cells. Plasma membrane, mitochondrial or lysosomal transporters are represented by green, gray or blue trapezoids, respectively. The transporters known to be crucial for cancer metabolism are named by their common names (see the main text); in brief from the top left to the bottom right: LAT1 mediates the exchange of nine EAAs in a Na^+^ independent mechanism and can be considered an harmonizer; ATB^0,+^ mediates the uptake of 18 out of 20 amino acids (AA^0,+^) with a Na^+^ and Cl^−^ dependent mechanism and can be considered a concentrative transporter; SNAT2 mediates the uptake mainly of Gln and Pro with a Na^+^ dependent mechanism and can be considered a concentrative transporter; ASCT2 mediates the exchange mainly of Gln and Ser with other neutral amino acids with a Na^+^ dependent mechanism and can be considered at a crossroad between concentrative transporter and harmonizer; SLC38A9 mediates the efflux of Gln and Leu, regulated by Arg, from lysosomes and is considered a “transceptor”; CAT generically indicates CAT1, CAT2 and CAT3 that mediate the uptake of Lys and Arg; xCT mediates the exchange of Glu and cysteine. Transport and enzyme reactions are represented by continuous arrows; enzyme or transport pathways are represented by dotted arrows. Mitochondrial or cytosolic cyclic pathways are depicted in green or blue, respectively; the urea cycle which is partly mitochondrial and partly cytosolic is depicted in the two colors. Amino acids are represented by the three letter codes and are depicted in blue except for the lysosomal (gray membrane) arginine, which is in gray indicating a lower involvement in mTORC1 recruitment; non-amino acid intermediates are depicted in black, energetic substrates are depicted in red, biosynthetic or signaling processes are depicted in dark red. The cellular glutamine pool is in a dotted box. DNA methylation (DNA-met); oxidized/reduced glutathione couple (GSSG/GSH); oxoglutarate (KG); oxaloacetate (OAA); branched chain amino acids (BCAAs); essential amino acids (EAA); all neutral and cationic amino acids AA(^0,+^); isocitrate (ICit); CAT stands for the CAT1,2 and 3 isoforms of transporters.

This finds an explanation in the key metabolic pathways in which these amino acids are involved, summarized in Figure 2. Eminent reviews extensively describe the amino acid metabolic pathways altered in cancer [1,2,3,12]. In the next sections, we briefly report some of the nodes in the most relevant pathways for cancer development and progression.

Then, we will focus on the role of specific membrane transporters, depicted in Figure 2, that form a network whose alteration allows cancer cells to rewire their metabolism.

## 2. Crucial Amino Acids for Cancer Cells

### 2.1. Glutamine and Glutamate

Glutamine is the most abundant amino acid in the human body with a plasma concentration ranging from 0.2 to 0.8 mM and an intracellular concentration ranging from 2 to 20 mM [13,14]. In physiological conditions, glutamine is involved in several cell processes: synthesis of amino sugar and nucleotides, ammonia scavenging, acid-base buffering in the kidney due to excretion of NH_3_ in the urine, ATP generation through the TCA and oxidative phosphorylation. Glutamine participates to the urea cycle in the liver and the gluconeogenesis in the liver and the kidney. Glutamine is also crucial for managing the redox balance in cells for different reasons: as an example, GSH synthesis can use glutamine-derived glutamate. Additionally, glutamine-derived α-ketoglutarate allows for NADPH production that, in turn, is important for maintaining the GSH/GSSG ratio. Finally, besides a metabolic role, glutamine is also responsible for gene expression regulation and cell signaling. Indeed, glutamine is involved in the activation of mTORC1 (Figure 2), the master regulator of the life/death balance in cells [13,14].

The great commitment of glutamine in the mentioned cell processes makes this amino acid “conditionally essential” in those physiological conditions, characterized by high proliferation rate, in which the endogenous glutamine synthesis is not sufficient to satisfy the cell need. This event occurs in stem cells and activated macrophages. In the context of cancer metabolism, glutamine becomes essential as well, for sustaining the metabolic rewiring. Indeed, glutamine participates in one of the most studied anaplerotic mechanisms in the context of cancer biology, that is, the entry of carbon skeleton into the TCA, as α-ketoglutarate with the consequent synthesis of ATP at the substrate level, in the reaction catalyzed by succinyl CoA-synthetase.

The mitochondrial utilization of glutamine starts with a reaction catalyzed by glutaminase, a crucial enzyme that, for this reason, represents an anticancer drug target [15,16]. The mitochondrial isoform of glutaminase is, however, a still-unsolved mystery concerning its actual localization: it seems plausible that glutaminase is in the mitochondrial matrix where glutamine is delivered by the activity of an inner membrane mitochondrial transporter whose identity remained unknown for decades [17]. Only very recently, a shorter isoform of the plasma membrane transporter for glutamine ASCT2 (SLC1A5) has been proposed as the mitochondrial glutamine transporter [18]. However, if glutaminase would be localized in the intermembrane space, a glutamine transporter would not be required [19].

Under canonical Warburg condition, the glutamine carbon atoms may undergo different fates depending on the rate of the TCA/oxidative phosphorylation [20]. Indeed, in some cancer types, ATP can be produced mostly by the cytosolic steps of glycolysis, then the TCA is slowed down and glutamine-derived α-ketoglutarate is exported into the cytosol for production of isocitrate, which is then converted to citrate for the synthesis of fatty acids required during cell proliferation for de novo membrane formation [21].

In other cancer types, the oxidative phosphorylation is more active since fatty acids are subjected to the β-oxidation, with the production of reducing equivalents for fueling the mitochondrial respiratory chain [22,23]. In this case, surprisingly, fatty acid synthesis and oxidation occur at the same time creating a futile cycle.

Furthermore, glutamine-derived glutamate in mitochondria is used for the synthesis of proline, a pathway interconnected also with the mitochondrial urea cycle [6], as depicted in Figure 2. The reaction catalyzed by glutaminase produces, besides glutamate, also nitrogen in the form of ammonia. This is, then, used for the asparagine synthesis from aspartate. It is important to note that glutamate can also be produced by glutamine for the synthesis of non-mitochondrial carbamylaspartate required for pyrimidine synthesis. In these conditions, cells are not exposed to toxic concentrations of glutamine-derived ammonia, while the metabolite dihydroorotate can be excreted in the blood where it can be easily converted to orotate further released in urine [24].

Glutamate is also used as a counter substrate for the accumulation of cystine from the external environment through the plasma membrane transporter xCT (SLC7A11). Cystine is then converted into cysteine for GSH synthesis for managing the redox stress (Figure 2). Finally, glutamate-derived glutamine serves as a source for alanine, aspartate and phosphoserine synthesis [6]. The complexity of the depicted scenario explains the involvement of several membrane transporters, reported in Figure 2, to manage the glutamine and glutamate flux in cancer cells which will be dealt with in Section 3.

### 2.2. Asparagine and Aspartate

The closely related amino acids asparagine and aspartate are connected with glutamine utilization in cancer. Intriguingly, circulating asparagine is mainly used for protein biosynthesis, rather than for intermediate metabolism. This is probably linked with the asparagine biosynthetic reactions that require high energy expenditure [5,25]. Notwithstanding, in cancer cells, the level of asparagine synthetase correlates with aggressiveness of tumors and, often, with poor prognosis indicating that asparagine is increasingly produced by cancer cells. In line with these observations, chemotherapy based on lowering asparagine blood levels is employed in some human lymphomas [26].

Besides the involvement in protein synthesis, asparagine has been proposed as a fundamental harmonizer of amino acids pool [25]. In other words, cells, through the activity of exchange transporters, such as ASCT2 (SLC1A5) (Figure 2), can release asparagine and absorb other amino acids, such as serine and glutamine, whose accumulation is required for accomplishing other cell pathways [19]. Furthermore, asparagine seems to reduce apoptosis induced by glutamine withdrawal in cancer cells by regulating the transcription of anti-apoptotic proteins [27].

In contrast to asparagine, aspartate circulating levels are low and its biosynthesis in cells relies on the activity of the enzyme glutamic oxaloacetic transaminase (GOT). Two isoforms of this enzyme, GOT1 and GOT2, localize in the cytosol or the mitochondria, respectively [6]. The synthesis of aspartate requires oxaloacetate and glutamate that can both derive from glutamine, implying that aspartate synthesis is connected with glutamine availability.

Cytosolic aspartate is used, besides for protein synthesis, for purine and pyrimidine synthesis, together with glutamine and glycine. These pathways are, obviously, strongly stimulated given that nucleotides are required for DNA/RNA production and, hence, for cancer cell proliferation (Figure 2) [28]. As an example, purine analogs are used in the therapy of several cancers, such as chronic lymphocytic leukemia [29].

### 2.3. Leucine and Other Essential Amino Acids

Besides the synthesis of proteins, the essential amino acids are involved in several cell pathways, some of which are summarized in Figure 1 and Figure 2.

Among the canonical ten essential amino acids, there is a small group constituted by leucine, valine, and isoleucine collectively known as branched chain amino acids (BCAAs). Notably, leucine is the most abundant amino acid in proteins, and therefore it is crucial in the signaling of the amino acids sufficiency to the lysosomal mTORC1 via the action of the SLC38A9 transporter, that is defined as “transceptor”, i.e., a transporter with a receptor function [30,31].

Another essential amino acid, arginine, is also involved in mTORC1 regulation even if with a secondary role with respect to leucine [31,32] (Figure 2). Furthermore, leucine has a role also in glutamine utilization in cancer because it acts as an allosteric regulator of glutamate dehydrogenase, the enzyme responsible for the conversion of glutamine-derived glutamate into α-ketoglutarate [33].

In terms of energetic metabolism, the essential amino acids give rise to diverse anaplerotic reactions fueling the TCA (Figure 1). In this respect, one of the key metabolites is Acetyl-CoA deriving from BCAAs, lysine, threonine, or tyrosine. Besides the employment in the TCA reactions for ATP, NADH, and FADH_2_ production, Acetyl-CoA deriving from essential amino acids is used for anabolic processes such as lipogenesis [34]. Indeed, the maintenance of a precise ratio among the different EAAs allows for a proper lipogenesis in adipocytes.

Moreover, Acetyl-CoA derived from EAAs catabolism is responsible for regulating gene expression through histone acetylation [35]. Therefore, some EAAs indirectly participate in cancer epigenetics.

One of the key proteins responsible for the flux of EAAs in cancer cells is the plasma membrane transporter LAT1 (SLC7A5) [36]. The regulation of gene expression can be ascribed also to another essential amino acid, arginine, whose flux is regulated by some of the membrane transporters depicted in Figure 2. Arginine is metabolized via arginase to ornithine used for the synthesis of putrescine, spermine and spermidine (Figure 2) [5,6].

These polyamines interact with DNA inducing chromatin remodeling. Furthermore, polyamines can act post-transcriptionally forming complexes with RNA, increasing RNA stability and, hence, protein synthesis efficiency [37]. However, one of the key metabolites deriving from arginine is the gasotransmitter nitric oxide (NO) through the reaction catalyzed by nitric oxide synthase (NOS) with the production of citrulline. Three different isoforms of NOS are present in humans, among which iNOS, i.e., inducible NOS [38].

Curiously, high levels of iNOS have been detected in several tumors [39] even if an unequivocal role of NO in cancer has not been defined yet. The increased production of NO from iNOS, reaching micromolar supraphysiological concentrations, has been linked with augmented protein nitrosation or nitrosylation that stimulates cell growth. Moreover, iNOS-derived NO may weaken the immune response by host T-lymphocytes stimulating the phenomenon of immune evasion typical of some cancers [40].

It has to be stressed, however, that the reaction catalyzed by iNOS oxidizes NADPH which is a precious cofactor for cancer cells to manage oxidative stress and to boost anabolic reactions. Therefore, it is plausible that NO production takes place only in those developmental stages in which it is strictly required.

Another essential amino acid with relevance to human health is tryptophan that, besides feeding the TCA through pyruvate and Acetyl-CoA, is responsible for regulating the immune response.

The reactions catalyzed by tryptophan 2,3-dioxygenase (TDO) and indoleamine 2,3-dioxygenase (IDO) produce kynurenine, a tryptophan metabolite able to increase the immune evasion during metastasis. In line with this fundamental role, IDO inhibitors have been designed as adjuvants of the current chemotherapy [41].

Finally, threonine, besides a role in Acetyl-CoA production, represents a link with the crucial one-carbon pathway discussed below. Degradation of threonine, indeed, replenishes the cellular pools of glycine and SAM [42].

### 2.4. Cysteine, Methionine, Serine, Glycine and the One-Carbon Atom Metabolism

The amino acids methionine, serine and glycine are involved in a pathway known as one-carbon (1C) metabolism that, in turn, is linked with cysteine synthesis required for GSH synthesis together with glutamate and glycine (Figure 2) [6,7,12]. In this pathway, a set of reactions give rise to the methionine cycle and to the folate cycle which are interconnected through a common metabolite, tetrahydrofolate, that is considered a universal one carbon acceptor [12].

This metabolism allows for the transfer of single carbon units from nutrients, mostly amino acids, to purine and pyrimidine synthesis, polyamine metabolism, DNA methylation, and cysteine synthesis. The complexity of the interconnections underlying 1C metabolism delayed the annotation of this pathway as a hallmark of human cancers. Interestingly, some early observations were collected in 1948 when children with acute leukemia, treated with folate-deficient diet, showed a reduction of leukemia cells indicating that folate cycle might be involved in cancer development and progression [43]. In line with these preliminary observations, several anticancer drugs, namely antifolates, have been designed to block 1C metabolism. However, given the role of THF in healthy tissues, several concerns arose around the side effects caused by these drugs [44]. To fulfil the 1C metabolism, three amino acids are crucial, serine/glycine for the folate cycle and methionine for the methionine cycle (Figure 2).

Serine is provided to cells mainly in two ways: i) it is avidly taken up from the external environment via plasma membrane transporters, such as ATB^0,+^ and ASCT2, and ii) synthesized from the glycolytic intermediate 3-phosphoglycerate in a three steps process requiring also glutamate [7,44] (Figure 2). Macropinocytosis phenomena were also proposed to be involved in serine absorption [44]. Therefore, endogenous synthesis of serine links the glucose and the amino acid metabolisms with important outcomes in cancer cells rewiring.

Serine is also used in the synthesis of sphingosine and phosphatidylserine [7]. An intriguing interplay is that between serine and glycine in the folate cycle, compartmentalized in both cytosol and mitochondria. The reactions run in two opposite directions (Figure 2). In the cytosol, serine is synthesized from glycine with NADPH utilization, while in the mitochondria, glycine is synthesized from serine with NADPH production crucial for the management of redox stress and proline synthesis [7,44].

Furthermore, in mitochondria the reaction that oxidizes formyl-THF to THF and CO_2_, produces NADPH increasing the pool of folate-derived NADPH. In cancer cells under high proliferation rate, the cytosolic reaction catalyzed by MTHFD1 may go in the opposite (mitochondrial) direction, producing NADPH for supporting cytosolic fatty acid synthesis, required as both signaling molecules and building blocks of membranes [44,45,46]. Interestingly, in cancer cell lines, serine starvation, coupled to increased levels of glycine in the medium, reduces cell proliferation [47,48] due to Me-THF depletion or to the absence of serine. For the sake of clarity, besides serine and glycine, other compounds fuel the folate cycle, namely choline, histidine, threonine and formate.

Quantitatively, choline, serine, and glycine are the most relevant donors [46] even if formate, produced in the mitochondrial side of the cycle, becomes the principal 1C donor for cytosolic reactions in highly proliferative cells [46]. Interestingly, formate is also produced by tryptophan degradation through another relevant amino acid-derived metabolite, namely kynurenine, as described above [7]. Taking into account that free formate in circulating blood is considered a toxin, generated by methanol poisoning, the folate cycle has also a role in detoxifying the organism [46].

While serine and glycine are crucially involved in the folate cycle, the essential amino acid methionine is crucial for protein synthesis and gives rise to the methionine cycle, the second half of the 1C metabolism pathway (Figure 2).

Methionine, mainly taken up via the essential amino acid transporter LAT1 [49] and the broad amino acid transporter ATB^0,+^ [50] serves mainly as methyl donor through the S-Adenosyl-methionine (SAM) active form for DNA methylation in the cycle, thus contributing to epigenetics. Interestingly, cancer cells often rely on SAM availability and, then, on the methionine cycle rate.

Increased SAM concentration gives rise to hypermethylation of DNA which normally triggers inappropriate gene silencing and histone methylation with aberrant cell growth [51,52]. S-adenosyl-homocysteine (SAH), produced from SAM after methyl transfer, is at a crossroad to produce methyl-THF or homocysteine. Methyl-THF enters the above described folate cycle, while homocysteine produces cysteine which is involved in GSH synthesis as previously described.

This last step is very critical in cancer cells survival given their peculiar need for controlling the GSH/GSSG ratio, i.e., the redox defense. Besides the synthesis of cysteine, the methionine-derived sulfur group is responsible also for H_2_S production, a gas transmitter with a variety of cell functions, among which is redox stress defense [53].

Finally, a methionine sensor in the cell has been recently described as responsible for nutrient sensing through mTORC1 [54].

### 2.5. Proline as the Bridge Between TCA and Urea Cycle

Proline is either synthesized by glutamate or taken up by transporter-mediated processes from the extracellular collagen that is enriched in proline and hydroxyproline. De novo synthesis occurs converting glutamine-derived glutamate to the key intermediate metabolite of proline metabolism that is pyrroline-5-carboxylate (P5C) also known as hydroxyproline. However, a fraction of proline biosynthesis relies on arginine-derived ornithine that is also converted to P5C through the action of the ornithine aminotransferase [7,55].

In the first case, the enzyme P5C reductase consumes NADPH. It is important to highlight that P5C is also the first product of proline catabolism through a redox reaction producing FADH_2_ that links proline to the oxidative phosphorylation. Then, P5C is further oxidized to glutamate that enters the TCA as α-ketoglutarate with a further synthesis of ATP and production of reducing equivalents for the oxidative phosphorylation (Figure 2).

Given the scenario depicted above, it is not trivial to consider proline as a bridge between TCA and urea cycle with great relevance for cancer rewiring (Figure 2). It is important to stress that in the context of cancer development, proline plays a unique role in tumor microenvironment influencing the remodeling of the extracellular matrix that is responsible for cancer aggressiveness and EMT [55,56].

Indeed, collagen is formed by a surprisingly high percentage of hydroxylated proline (up to 25% of the total amino acid composition). The vitamin C-dependent enzyme prolyl-4-hydroxylase is overexpressed in several cancers because changes of ECM mechanical properties, via collagen deposition, promote invasiveness and metastasis [57].

Very interestingly, a role for proline-derived collagen in terms of epigenetic regulation has been recently proposed. It has been shown that following an increase of collagen synthesis dependent on proline viability, the activity of the hydroxylating enzyme consumes vitamin C that is, then, depleted from the cellular pool. This, in turn, negatively affects the DNA demethylases with the consequent increase of DNA methylation. This phenomenon has been described in embryonic stem cells as well as in aggressive breast cancer [58]. Proline taken up from collagen degradation is used for energy production in TCA.

Given that collagen may incorporate a great amount of proline/hydroxyproline, it has been proposed that tumors may use collagen synthesis as a temporary “dump” of reducing equivalent in the form of proline [55].

### 2.6. Aspects of Amino Acids in Cancer Nutrition

Nowadays, an additional issue has been linked to cancer, that is nutrition, which has also become a fashionable topic for the nonscientist community [59]. In other words, how could dietary modification as a supplement for conventional cancer therapy improve the treatments and/or reduce the side effects of the therapy?

This practical approach is receiving growing attention due to sizable pieces of evidence that the manipulation of cancer metabolism may be driven by modifications of microenvironment composition in terms of nutrients availability. The main effects of such a strategy would be the enhancement of drug efficacy, the activation of anticancer immune response, the supply of metabolites specifically toxic for cancers, and the induction of tumor starvation.

## 3. Amino Acid Transporters in Cancer

The demand of amino acids by cells calls for a synergistic and finely regulated action of plasma membrane transporters that, in physiological conditions, allows for proper absorption and distribution of these nutrients to tissues as well as for the reabsorption from kidney ultrafiltration. This network, that guarantees homeostasis and homeorhesis [60], becomes even more relevant in the context of cancer metabolic rewiring characterized by alternative and, in some cases, paradoxical utilization of some amino acids [1,2,3,4].

The scenario depicted in Figure 2 is representative of those amino acid transporters, whose expression/function is altered in some human cancers (Table 1). Each transporter will be described in the following paragraphs in terms of the role played in the rewired cancer metabolism.

It has to be stressed that one feature of amino acid transporters is their redundancy. In other words, the same amino acid can be transported by more than one protein and the same protein can mediate the traffic of more than one amino acid.

Besides the complex interplay with different tumor microenvironment, the transporter redundancy is at the basis of the great diversity of the transporter collection in the different human cancers. Indeed, each cancer develops a specific layout of expressed and overexpressed amino acid transporters as a consequence of hormonal, environmental, and epigenetic stimuli [5,6,7,10].

In the specific field of amino acids, a lot of information is now available on the relationships between the metabolic rewiring of cancer cells and the layout of amino acid transporters expression/function [5,6,7].

In the following sections, an overview on the link between membrane transporters and human cancers will be given with a special focus on those proteins responsible for mediating the traffic of the most relevant amino acids for cancer rewire.

### 3.1. SLC1A5: Role in the Traffic of Glutamine and Other Neutral Amino Acids in Cancer

SLC1A5 referred to as ASCT2, is a plasma membrane transporter with a quite broad tissue distribution that underlies its role as “harmonizer” of amino acid pools in the human body [113,136].

The name ASCT2 is an acronym standing for AlaSerCysTransporter 2 according to the substrate specificity described for this protein after preliminary observations conducted using different experimental models: intact cells [137], *Xenopus* oocytes [138], and proteoliposomes reconstituted with the transporter extracted from rat kidney, human cell lines or produced by heterologous expression in *P. pastoris* [139,140].

Subsequently, it was demonstrated that, despite the name, the main substrate of ASCT2 is glutamine, while cysteine acts as a regulator rather than a substrate [141]. ASCT2 catalyzes a peculiar three substrates reaction in which the antiport of amino acids is coupled to the transport of at least one sodium ion from the extracellular to the intracellular environment. The transport is functionally asymmetric. In other words, Km on the internal or external side of the protein differ of at least one order of magnitude [140]. Glutamine, serine, asparagine, and threonine are bidirectionally transported while alanine, methionine and valine are only inwardly transported.

Interestingly, ASCT2 recognizes, at a lower affinity, the negatively charged amino acids glutamate and aspartate [142,143,144]. The transport cycle involves, in addition to sodium, also a proton and can be considered, plausibly, a reminiscence of the kinship with the high-affinity glutamate transporters SLC1A1-3 and SLC1A6-7 [145].

An intriguing novelty recently emerged on ASCT2 biology, i.e., the mitochondrial localization of a shorter splicing isoform of this transporter. This finding may have relevant outcomes in physiological and pathological conditions in which alternative glutamine utilization is required since this isoform is also over-expressed in cancer [18].

The 3D structure of ASCT2 revealed a homotrimeric assembly and a fold very similar to that of the bacterial glutamate transporter Gltph [146].

ASCT2 falls in the elevator mechanism group of transporters. Plausibly, the three monomers work independently from each other, giving rise to the previously detected random simultaneous mechanism [147].

ASCT2 physically interacts with cholesterol, which modulates the ASCT2 transport activity. Two N-glycosylation sites are involved in trafficking ASCT2 to the plasma membrane [148]. In the last decades, several reports highlighted the overexpression of ASCT2 in virtually all human cancers (Table 1).

Indeed, cancers are characterized by a phenomenon known as “glutamine addiction” according to which glutamine endogenously synthesized is not sufficient for cell metabolism and signaling and must be taken up from external environment for accomplishing all the pathways above mentioned [3,4,28].

The described transport cycle of ASCT2 has some implications: the sodium gradient directed towards intracellular space drives the transport together with the gradients of the counter-transported amino acids (Figure 2). Therefore, the overall transport reaction is electrogenic due to the coupling of an electroneutral amino acid exchange with sodium transport [140].

When amino acids are required as a carbon source for energy metabolism, the preferred reaction is the net uptake of the C5 amino acid glutamine, coupled to the efflux of a C3-C4 amino acid such as serine or asparagine, allowing oxidation of one of the carbon atoms of glutamine in the TCA (Figure 1) [2,19].

It has to be stressed that, besides glutamine, ASCT2 may take up also serine, which is strongly required by cancer cells for managing redox stress when the synthesis from glucose or glycine is not sufficient (Figure 2) [140].

The regulation of ASCT2 expression is under investigation since 2004 when it has been demonstrated that the glutamine availability, per se, regulates the expression of this transporter through a mechanism involving the transcription factor dimer FXR/RXR [149]. Then, some studies have been conducted in non-cancer models and positive regulation by EGF and aldosterone and negative regulation of ASCT2 expression by leptin in the intestine have been shown, [150].

A greater number of studies has been conducted in cancer cell models showing that (i) loss of the tumor suppressor pRb causes the over-expression of ASCT2 through E2F-3 transcription factor [151], (ii) the proto-oncogene c-Myc induces overexpression of ASCT2 through a responsive element on ASCT2 promoter [152]. A more controversial role can be described for mTOR. In some studies, mTOR has been shown as a regulator of ASCT2 transport activity, while in others it has been reported that mTOR signaling is not affected by ASCT2 and vice versa [136].

This discrepancy may be explained by the extreme variability of cancer cell metabolism. Thus, a different response to the same stimulus is plausible in different cancer types or even in same cancer during different stages of differentiation.

The key role of ASCT2 to sustain cancer cell metabolism has been demonstrated by silencing ASCT2 in different cell lines, with the consequent reduction of both cell viability and of downstream glutamine-linked pathways [9,61,113,153,154,155]. These observations represented the starting point for pharmacological studies devoted to identifying molecules able to specifically target ASCT2 in cancer cells for reducing growth and/or aggressiveness (see conclusions).

### 3.2. SLC6A14: Concentrative Transporter for Amino Acids in Cancer

SLC6A14 referred to as ATB^0,+^ is a plasma membrane transporter mainly expressed in kidney, intestine and mammary glands [50,61]. The gene encoding ATB^0,+^ has been isolated and cloned in 1999 and its functional and kinetic characterization has been performed in intact cell systems [156]. As stated in the introduction, one of the features of amino acid transporters is their polispecificity. In this respect, ATB^0,+^ represents the best example since it recognizes 18 out of 20 proteinogenic amino acids.

Amino acids are transported in a strictly dependent Na^+^ and Cl^−^ reaction (Figure 2). In particular, two Na^+^ and one Cl^−^ ions are taken up, together with one amino acid. Even if these basic features have been described since the early studies, the functional and kinetic characterization of ATB^0,+^ is not yet completed. Indeed, the Km of ATB^0,+^ has been reported only for few substrates, such as glutamine, being in the low millimolar range, suggesting that this protein is a low-affinity transporter. Despite this characteristic, ATB^0,+^ is a high capacity transporter, given its dependence by the ion gradients across the plasma membrane that drive the intracellular accumulation of amino acids even in the presence of an unfavorable amino acid concentration gradient [157].

The very broad substrate specificity of ATB^0,+^ allows cells to satisfy their needs under different metabolic conditions [50,61]. This feature becomes even more relevant in the context of cancer metabolic rewiring [9,155]. The possibility of transporting different substrates allows cells to derive nutrients from the tumor microenvironment (Figure 2). Given the above premises, it is not a surprise that ATB^0,+^ is upregulated in several human cancers (Table 1), originating even from tissues that normally do not express this protein [9]. This indicates that ATB^0,+^ may be used by cancer cells to adapt to the changing growth conditions and, then, to the energetic fuels [19].

ATB^0,+^ overexpression has been linked to the massive uptake of glutamine typical of the “glutamine addiction” phenomenon. However, the role of ATB^0,+^ needs to be deepened considering its ability to mediate the uptake of other amino acids that are also required by cancer cells. Then, in the context of cancer metabolic rewire, ATB^0,+^ may be involved in (i) methionine uptake to sustain DNA methylation; (ii) arginine supply required for regulating angiogenesis through NO synthesis; (iii) proline accumulation from the surrounding collagen to regulate TCA cycle and epigenetic phenomena.

Therefore, ATB^0,+^ may be interpreted as a “factotum” that cooperates with transporters characterized by a more specific substrate recognition.

Last but not least, a unique feature of ATB^0,+^ is its ability to mediate the transport of carnitine that may also be required by some cancers that depend on fatty acid oxidation [157].

The above-described wide collection of possible substrates has not yet been deeply investigated in terms of structure/function relationships given that the 3D structure of this protein is still not available. Therefore, the molecular determinants for substrate recognition are still unknown. 

However, the recently solved 3D structure of SLC6A19 [158], that belongs to the same family, may furnish some clues to improve the ATB^0,+^ homology model as it has been recently described by a computational approach [159].

The regulation of ATB^0,+^ expression/function is not yet fully deciphered even though some studies are available concerning the role played by collectrin in the kidney and ACE2 in the intestine for the trafficking at the plasma membrane. Furthermore, leptin seems to be related to an increased expression of ATB^0,+^ as well as EGF and GH, even though the mechanisms are not described yet [155].

Furthermore, it has been reported that the phosphorylation of ATB^0,+^ by PKC has a positive effect on its transport activity [160]. Finally, an aspect that deserves attention is the employment of ATB^0,+^ as a vector of anticancer drugs, in the form of amino acid-linked prodrugs [161].

### 3.3. SLC7A1, SLC7A2 and SLC7A3: Role in the Traffic of Cationic Amino Acids in Cancer

SLC7A1, A2, and A3 referred to as CAT1, CAT2, and CAT3, respectively, are plasma membrane transporters that form the subfamily CAT in the SLC7 family. This branch includes also another transporter, SLC7A4 referred to as CAT4 that, however, seems not to share the same transport properties with CAT1, 2 and 3. In contrast with the branch of LATs, the CAT members are glycosylated and do not associate with an ancillary protein.

Different tissue distribution and subcellular localization have been provided for the three CATs. In particular, CAT1 is ubiquitously expressed, except that in the liver and lacrimal gland; in polarized epithelia, CAT1 is mainly localized in the basolateral membrane but also the intracellular membranes. CAT2 exists in two splicing variants namely CAT2-A and CAT2-B which have different tissue expression. Indeed, CAT2-A is mainly present in the liver, the skeletal muscle and the pancreas, while CAT2-B is inducible in several cell types. CAT3 has a narrower tissue distribution being expressed in the thymus, the ovary, the testis, and the brain. The four above-mentioned proteins have been studied in intact cell systems, *Xenopus* oocytes, and animal models [162,163]. These transporters show a strong preference towards the proteinogenic cationic amino acids arginine and lysine, as well as the non-proteinogenic amino acid ornithine (Figure 2).

Apart from the Na^+^-independence, the transport cycle shows different mechanisms and affinities depending on the transporter. In particular, CAT1 exhibits Km values for arginine, lysine and ornithine, in the micromolar range. Moreover, the uptake of amino acids is trans-stimulated by intracellular substrates and by hyperpolarization [164,165]. CAT2-A, CAT2-B and CAT3 are less sensitive to the *trans-stimulation* [164]. Interestingly, CAT2-B and CAT3 have a slightly lower affinity towards substrates with respect to CAT1, while CAT2-A has a tenfold lower affinity towards substrates if compared to CAT2-B [164].

A slight pH dependence has been demonstrated for CAT2-B, while CAT1 and CAT3 are mostly insensitive to pH variations in the range of 5.5–8.0. The only exception is the transport of histidine, that even if it is not a “canonical” substrate of CATs, it is recognized by CAT1 at acidic pH, i.e., when histidine is in the protonated form. No 3D structure is available for these transporters. However, substrate specificity studies together with competition experiments highlighted that CATs prefer cationic amino acids with longer carbon backbone. Indeed, the affinity decreased from arginine to ornithine [162]. Furthermore, a key aspartate residue has been identified as responsible for substrate recognition and gating, that is conserved in all SLC7 members, except for LATs.

An interesting structural difference between LATs and CATs is the number of transmembrane domains that are 12 and 14, respectively. This difference underlies a different evolutionary history. 

The two extra segments have originated from a duplication event that occurred in the two last transmembrane segments of a protein being the common ancestor of LATs and CATs [163]. The overlapping substrate specificity and the transport mechanism of the four transporters correlate with the crucial role of arginine and lysine in cell physiology. These are two essential amino acids which, besides the involvement in protein synthesis, are also required for other cell pathways as highlighted in Figure 1 and Figure 2.

In particular, arginine is a substrate for NO synthesis, responsible for the inflammatory response [38]. The apparent redundancy of CATs well correlates with the compensative phenomena observed in KO mice for CAT1. These transgenic mice express CAT3 with, as a consequence, a relatively normal fetus development [166].

The studies of CATs regulation is still at infancy: a translational control has been proposed for CAT1 by the microRNA miR-122 responsible for its down-regulation in the liver [167]. Moreover, activation of PKC promotes ubiquitination of CAT1 for its recycling [163].

Given the described features, it is plausible that the roles played by CATs in the context of metabolic rewire are (i) allowing for arginine and lysine accumulation to sustain anaplerotic reactions of TCA (Figure 1); (ii) providing cells with arginine for NO synthesis; (iii) furnishing ornithine to cells to be used in polyamine synthesis (Figure 2). Recently, an interesting link of glutamine and arginine to cancer cells was also described. It has been shown that p53 can induce CAT3 expression in cancer cells upon glutamine starvation to restore cell viability via mTORC1 activation [168]. In line with this, CAT1, CAT2, and CAT3 are deregulated in several human cancers (Table 1).

### 3.4. SLC7A5: Role in the Traffic of Essential Amino Acids in Cancer

SLC7A5 referred to as LAT1, is a plasma membrane transporter with a quite narrow tissue distribution being mainly expressed in the brain, the placenta, the testis and the bone marrow [36,163]. The LAT1 expression in epithelia underlies its polarized subcellular localization. Indeed, LAT1 is expressed in basolateral membranes except in the case of the blood-brain barrier where the protein localizes both at the apical and basolateral membrane [169]. Furthermore, in the placenta LAT1 has been found on both the maternal and fetal side of the barrier [170]. More recently, subcellular localization in the lysosomal membrane has also been proposed [171].

LAT1 is classified in the group of the heterodimeric amino acid transporters (HATs). These proteins are characterized by the formation of functional heterodimers between one light chain, belonging to SLC7 family, and one glycoprotein belonging to the SLC3 family [163]. In particular, LAT1 is associated with SLC3A2, referred to as CD98 or 4F2hc, via a covalent bond occurring between two conserved cysteine residues of the two proteins. The studies on LAT1/CD98 heterodimer have been conducted in cell systems [49,163] and in proteoliposomes reconstituted with human isoforms of the two proteins forming the heterodimer [172].

The first studies conducted in intact cell systems demonstrated that LAT1/CD98 heterodimer is responsible for mediating the uptake of nine essential amino acids, cysteine, and at a lower extent, glutamine [49,163]. LAT1 is also involved in the transport of non-amino acid substrates such as the thyroid hormones T3 and T4, and the dopamine precursor L-Dopa [36]. The ability to recognize essential amino acids and to provide the brain and the placenta with them makes this protein a crucial player in organism development as testified by the observation that KO embryos for LAT1 are not vital [170].

LAT1 is also able to recognize some drugs such as gabapentin as a substrate [36]. LAT1 malfunctioning has been linked to a familiar form of autism spectrum disorder due to the great reduction of essential amino acids in the brain [173,174].

LAT1 expression is reduced in intrauterine growth restriction, a clinical condition responsible for perinatal complications linked to a reduced presence of leucine and phenylalanine in the placenta [175].

In the last decade, experiments conducted with proteoliposomes revealed that LAT1 is the sole component of the heterodimer involved in the transport function, while CD98 has an accessory role, linked to chaperoning the protein to the definitive location in the plasma membrane [172,176], even if the molecular mechanism is not described yet.

This is in line with the pleiotropic functions of CD98 ranging from the interaction with other members of the SLC7 family (see Section 3.5) to the activation/regulation of cell pathways which are independent on amino acid transport and metabolism [177]. The transport cycle of LAT1 is a Na^+^ independent antiport of amino acids. Thus, LAT1 is a “harmonizer” of amino acid pools, rather than a concentrative transporter.

Then, the driving force for exchanging amino acids through LAT1 derives by the counter-gradients of substrates across the plasma membrane [36]. The transport is functionally and kinetically asymmetric: the majority of amino acids is only inwardly transported, except for histidine, tyrosine and, at a lower extent, glutamine which are bidirectionally transported [178]. In terms of kinetics, the external side of the transporter shows a Km range from the micromolar to the low millimolar, while on the internal side, millimolar range Km values have been measured [178].

The mentioned studies allowed for the identification of the minimal requirements for a molecule to be transported by LAT1 in a pre-structure era: two vicinal carboxylic and amino groups, a large and hydrophobic side group [36]. These observations have been confirmed by structure/function relationships studies conducted using site-directed mutants and, then, by the recently solved 3D structure of the LAT1/CD98 heterodimer by CryoEM [179,180]. The transport cycle of LAT1 is, indeed, regulated by a key gating residue, i.e., Phe252, that is conserved also in the bacterial ortholog AdiC of *E. coli* [178]. LAT1 physically interacts with membrane cholesterol that seems to have an influence on the transport rate rather than on the affinity of L-DOPA and leucine [179,180,181].

The regulation of LAT1 expression has been also a matter of investigations in the last years even if it is still an *in nuce* field. Glucose and insulin levels modulate LAT1 expression in different tissues, and this is crucial for diabetic patients [182]. Increased plasma glucose level induces down-regulation of LAT1 expression in the muscle. Low plasma glucose level triggers the upregulation of LAT1 in the retina [183]. Low circulating insulin induces up-regulation of LAT1 in the muscle., Low LAT1 expression in β-cells causes a reduction of insulin synthesis due to the lack of amino acids, such as leucine, highly represented in the sequence of insulin [184]. Furthermore, the promoter region of LAT1 hosts responsive elements for the proto-oncogene c-Myc, the hypoxia-inducible factor 2 HIF2, for RXRa, and YAP/TAZ [36]. Interestingly, these transcription factors are responsible for regulating cell cycle and cell metabolism.

Therefore, even if a complete scenario is not available, the described data furnishes a solid basis to explain the strong up-regulation of LAT1 in virtually all human cancers (Table 1) according to GENT database [185]. As in the case of ATB^0,+^, LAT1 is over-expressed also in cancers originating from tissues in which the protein is not at all present in physiological conditions [1,9]. Moreover, LAT1 expression in cancer is considered a prognostic factor of metastasis [186]. It is interesting to highlight that the regulation by transcription factors involved in both amino acid and glucose homeostasis suggests the existence of a complex interplay between different nutrients required by cells in either physiological or pathological conditions.

Taken together, all the above observations, the main function of LAT1 in the context of cancer rewiring should be that of mediating the uptake of (i) leucine for the regulation of protein synthesis, mTORC1 signaling and glutamine utilization; (ii) methionine for DNA methylation and methionine cycle; (iii) BCAAs for Acetyl-CoA production and then lipogenesis and/or ATP production (Figure 2).

It is important to stress that the preference towards histidine as internal counter substrate, implies that this essential amino acid may be released to increase the accumulation of other amino acids required for other functions rather than merely protein synthesis. That histidine is the preferred efflux substrate of LAT1 has been definitively demonstrated by conditional mice knockout of LAT1 that accumulate histidine in the brain [173].

In line with the key role played by LAT1 in human cancers, its silencing induces lower cell viability in different models with alteration of downstream pathways, such as that of mTORC1 that senses, besides others, the leucine levels in cells [187,188]([36] and refs therein). As in the case of ASCT2, these observations made LAT1 a very interesting druggable target for anticancer therapy (see conclusions).

### 3.5. xCT: Role in Managing the Redox Stress of Cancer Cells

SLC7A11 referred to as xCT, is linked by a covalent disulfide bond to the ancillary subunit SLC3A2 (also known as CD98 or 4F2hc) as LAT1. xCT is responsible for the transport activity of the system while CD98 is a chaperone ensuring both correct localization at the plasma membrane level and protein stability of xCT [176,189].

xCT plays a central role in providing cysteine for the biosynthesis of GSH. Indeed, a great amount of cysteine is exported from the liver but this amino acid is rapidly oxidized to cystine in the extracellular environment where reaches approximately 50 µM [190]. Cystine is taken up by xCT that mediates a Na^+^ independent antiport of cystine in the exchange for glutamate with 1:1 stoichiometry [191,192]. Since the intracellular pool of cystine is negligible because it is rapidly converted to cysteine and the intracellular glutamate concentration is higher than the extracellular one, the driving force for xCT transport is represented by the concentration gradients of the counter-substrates [192].

Besides the role in cysteine and GSH homeostasis (Figure 2), xCT seems to play an important function also in oxidative stress response. Moreover, a close association between ferroptosis, a recently discovered form of cell death, with cystine depletion following xCT impairment was described [193].

Ferroptosis is a non-apoptotic cell death mechanism due to the over-accumulation of lipid hydroperoxides in an iron-dependent manner. Lipid hydroperoxides accumulation in cells can be avoided thanks to the action of the glutathione peroxidase 4 (GPX4). This enzyme converts lipid hydroperoxides to alcohols using GSH with reduction of ferroptosis [194].

In line with this, the pharmacological inhibition of xCT activity by drugs such as erastin results in depletion of intracellular GSH and induction of ferroptotic cell death [194]. Furthermore, sulfasalazine inhibits xCT with consequent sensitization of cancer cells to proteasome inhibition [195].

Moreover, many studies have documented that xCT protects cells from several stresses. As an example, the upregulation of xCT in neuronal and cancer cell lines confers resistance to the oxidative stress and renders cancer cells more resistant to chemotherapy [196,197].

In the light of the established pro-survival function of xCT under stress conditions, it is not surprising that this transporter is overexpressed in several tumors (Table 1). xCT expression is associated with poor survival in patients with malignant glioma and hepatocellular carcinoma [77,129]. Increased xCT expression is also a predictor of disease recurrence in patients with colorectal cancer [73]. In breast cancer, oncogenic *PIK3CA* revealed able to decrease cysteine uptake by both transcriptional and post-translational inhibition of xCT [198]. Surprisingly, in the condition of glucose starvation, xCT promotes cell death. This effect has been demonstrated in various cancer types, including breast, cervical, kidney, and brain cancers, as well as mesothelioma [199]. Two hypotheses have been put forward to explain this phenomenon. The first one moves from the transport cycle of xCT that causes a lowering of intracellular levels of glutamate which cannot be used to supply the TCA under glucose starvation. Then, cancer cells expressing a high level of this transporter are more dependent on glucose for survival. In line with this, the supplementation of α-KG in the medium of cancer cells overexpressing xCT, abrogates the cell death induced by glucose starvation. On the contrary, in the presence of xCT knockdown, the inhibition of the conversion of glutamate in αKG re-sensitizes cancer cells to glucose starvation.

The second hypothesis proposes that cystine uptake, rather than glutamate export, is the link between xCT and cell death induced by glucose starvation [199]. xCT seems to be involved also in glutamine dependency and glutaminase inhibition sensitivity of some cancers. As an example, the basal and claudin-low triple- negative breast cancer (TNBC) cells exhibit higher levels of xCT, greater cystine consumption and higher sensitivity to glutamine deprivation than other breast cancers and normal mammary gland epithelia. Such data suggest that TNBC consume glutamine to maintain xCT-mediated cystine/glutamate exchange, resulting in a marked glutamine dependency [200].

It is important to know that high levels of cystine in standard cell culture media render cells more dependent on glutamine and more sensitive to glutaminase inhibition [201]. It is known that glutamate inhibits glutaminase activity, therefore, it was suggested that high levels of extracellular cystine deplete intracellular glutamate through xCT-mediated cystine/glutamate exchange, resulting in glutaminase activation and glutamine dependency [202]. The variation of the xCT expression level in response to stress, such as oxidative stress and amino acid deprivation, are mediated by the nuclear factor erythroid 2-related factor 2 (NRF2), the activating transcription factor 4 (ATF4) and p53.

NRF2 is a master transcription factor that mediates antioxidant response. Under basal conditions, NRF2 is targeted by kelch-like ECH-associated protein-1 (KEAP1) and degraded through the proteasome. Oxidative stress induces oxidation of the reactive cysteine residues on KEAP1, resulting in the impairment of NRF2 degradation. NRF2 translocates into the nucleus where binds to antioxidant response elements (AREs) in the promoter regions of xCT gene [203]. ATF4 is a transcription factor that regulates redox homeostasis, amino acid metabolism, and endoplasmic reticulum (ER) stress. Under basal conditions, ATF4 mRNA translation is repressed. This repression is abrogated by the phosphorylation and by the inhibition of eukaryotic initiation factor 2α (eIF2α).

Phosphorylated eIF2α inhibits the transcription of ATF4 repressors, triggering the increase of ATF4 protein level. The kinases that catalyze eIF2α phosphorylation are activated by various types of cellular stresses, such as amino acid deprivation, ER stress, and viral infection. One of these kinases is the general control non-derepressible-2 (GCN2) that is activated by free tRNAs under amino acid deprivation. Subsequently, ATF4 binds to amino acid response elements in the xCT promoter [204].

Both ATF4 and NRF2 upregulate xCT while the tumor suppressor p53 represses the expression of this transporter, even if the mechanisms by which p53 regulates the xCT gene under stress condition remain unclear [205]. The levels of the xCT mRNA are also regulated by post-transcriptional mechanisms such as nonsense-mediated mRNA decay which is a surveillance pathway that targets mRNAs encoding proteins involved in stress responses. In the case of amino acid deprivation, NMD is inhibited resulting in xCT mRNA stabilization, increased protein levels, enhanced cysteine transport and GSH synthesis [206]. Some microRNAs downregulate xCT transcription in various human cancers leading to the xCT overexpression [207,208,209]. The transport activity of xCT seems to be modulated by the mammalian target of rapamycin complex 2 (mTORC2); the N-terminal serine 26 of xCT is phosphorylated by mTORC2 in response to growth factor stimulation, causing transporter function inhibition [210]. Given all the mentioned observations, it is plausible that xCT overexpression in human cancers (Table 1) has the main role in managing cell redox stress.

### 3.6. SLC38A2: Concentrative Transporter of Glutamine and Other Neutral Amino Acids in Cancer

SLC38A2 referred to as SNAT2, is a plasma membrane transporter belonging to the SLC38 family that includes 11 members. In particular, SNAT2 has been historically classified in a small group of transporters called system A [211,212]. This old but still used classification is based on the common transport features shared by the members of system A, that is the co-transport of extracellular Na^+^ downhill its concentration gradient across the plasma membrane, and the inhibition by the amino acid analogue MeAIB [213].

SNAT2 is ubiquitously expressed and, in polarized epithelia, it is localized in the basolateral membrane. The studies on SNAT2 have been conducted in cell system and *Xenopus* oocytes reaching a functional and kinetic characterization: the transporter can recognize glutamine, alanine, asparagine, cysteine, glycine, histidine, methionine, proline, and serine with Km values varying in the micromolar range. The stoichiometry of co-transport with Na^+^ is 1:1. The net uptake of a positive charge per each transport cycle is responsible for electrogenicity [214,215].

The 3D structure of SNAT2 is not available, therefore, homology models have been built on the basis of the proton-amino acid transporter ApcT from *M. jannaschii* revealing a LeuT fold [216].

Residues responsible for sodium and substrate binding have been identified by site-directed mutagenesis coupled to transport assays [215]. Moreover, the presence of a disulfide bridge in an extracellular loop of SNAT2 has been shown with the plausible role of sensing the cell redox potential [217].

The ubiquitous expression of SNAT2 makes this transporter crucial in several cell pathways and processes. As an example, SNAT2 is specifically expressed in GABAergic and glutamatergic neurons [218], playing a role in the glutamine/glutamate cycle [212]. A further functional cross talk takes place in the pancreas where SNAT2 is expressed in α-cells and another transporter, called SNAT3, is expressed in β-cells. The flux of glutamine between the two transporters and, then, between the two cell types in the pancreas, is responsible for regulating glucagon secretion for glucose homeostasis [219].

Moreover, SNAT2 is expressed in the placenta where is involved in providing the fetus with amino acids required for normal development and energy metabolism. Interestingly, SNAT2 in embryonic stem cells is responsible for proline uptake required for mTOR activation and differentiation stimuli.

Despite the ubiquitous tissue distribution, the expression of SNAT2 is not that of a housekeeping gene, but it is strongly regulated, in line with the mentioned pleiotropic roles. The substrate availability is the strongest regulatory signal of SNAT2 expression: amino acids deprivation, indeed, increases SNAT2 expression thanks to the presence of an amino acid response element (AARE), in the first intron of its gene [220]. In this context, SNAT2, like its companion SLC38A9, acts also as a transceptor being involved in sensing amino acid sufficiency in cells [212]. Moreover, glucagon up-regulates SNAT2 expression in the liver as well as in hypertonically stressed cells, suggesting a role for SNAT2 in the regulation of cell volume [221].

Considering all the above observations, it is not a surprise that SNAT2 is over-expressed in several cancers, as reported in Table 1. The roles played by SNAT2 in cancer rewiring may be various: (i) providing cells with proline for epigenetic control; (ii) providing cells with glutamine for energy metabolism; (iii) providing amino acids for signaling purposes merging the function of SLC38A9 in the lysosomal membrane; (iv) accumulating amino acids for protein synthesis and energy metabolism due to the concentrative transport mechanism (Figure 2). These findings suggest also SNAT2 as a potential drug target considering that its expression in cancer is strongly stimulated when ASCT2 silencing is performed [113].

## 4. Conclusions

The role of membrane transporters as drug targets is nowadays well assessed due to the location of these proteins at the boundary between the external and internal environments of cells. In this respect, transporters that are over-expressed in human cancers are particularly attractive as druggable targets for anticancer drugs. Indeed, the amino acid transporters dealt with in this review are the subject of active investigations to find potent inhibitors able to perform chemical silencing. This approach would overlap the silencing achieved by cell biology approaches that, generally, revealed useful in impairing the cancer cells viability.

For some of these transporters, namely ASCT2 and LAT1, the studies are at a more advanced stage [222,223]. Indeed, the molecule V-9302 showed anticancer activity in preclinical models also in combination with an inhibitor of glucose metabolism [224,225]. Furthermore, the drug JBH203 is the first-in-human phase I study of LAT1 inhibitor that has been tested in advanced solid tumors [226].

This is due to both the availability of the 3D structure, recently solved, and the well assessed experimental models for transport assay and functional characterization.

The other transporters are under active investigation and, plausibly, they will be better characterized in the near future.

A general flow chart for the identification of potent inhibitors consists in a first phase of *in silico* study in which a large-scale virtual screening of chemical interactors is performed. Then, the best hits predicted as inhibitors are validated by dose-response analysis of inhibition in the cell or artificial experimental models in which transport assay can be accurately measured. Using such a strategy in the case of LAT1 and ASCT2, inhibitors with different degrees of potency and specificity have already been identified which can be classified as substrate-analogs and covalent inhibitors ([36,136,227] and refs therein).

However, it has to be stressed that, in the last decades, it emerged that the simple view of a cancer blocking strategy based on one drug targeting one transporter is far from being true. Indeed, tumor plasticity allows cancer cells to overcoming the silencing of one membrane transporter by using other proteins, which are normally not expressed [153]. This phenomenon, of course, calls for a careful and rational drug design approach that takes into account several parameters, including the ability of cancer cells to adapt to their “nutrient” environment.

Therefore, these pioneering results let us hope that the current understanding of the biochemical features of cancer cells, together with the increasing knowledge of the structure–function relationships of the transporters, will open a new era in drug design for the development of novel and more effective anticancer drugs moving from the rewiring of cancer cell metabolism.

## Figures and Tables

**Table 1:** Expression of membrane transporters in human cancers.

*SLC1A5* (ASCT2)	*SLC6A14* (ATB^0,+^)	*SLC7A1-2-3* (CAT 1-2-3)	*SLC7A5* (LAT1)	*SLC7A11* (xCT)	*SLC38A2* (SNAT2)	References
PCA	PCA		PCA	PCA		[19,61,62,63,64,65,66,67]
CRC	CRC	CRC (C1-2)	CRC	CRC		[19,61,67,68,69,70,71,72,73,74,75]
HCC		HCC (C1)	HCC	HCC		[61,69,76,77]
LC			LC	LC		[19,61,67,78,79,80,81]
BC	BC	BC (C1)	BC	BC	BC	[19,26,61,62,67,69,79,82,83,84,85,86,87,88,89,90,91]
N & G			N & G			[61,92,93]
EC			EC			[61,94,95]
OC			OC		OC	[19,61,96,97]
RCC			RCC			[19,61,98,99]
	P & BC		P & BC	PC	PC	[61,100,101,102,103,104]
GC		GC (C1)	GC			[61,105,106,107,108,109,110]
			BT			[111]
		PM (C1)				[61]
CC	CC		CC		CC	[19,86,112,113]
OSCC			OSCC	OSCC		[114,115,116,117]
		TC (C1)				[118]
		M (C1)		M		[119,120]
		L (C1-2)	L			[121,122,123,124]
ESCA			ESCA	ESCA		[115,125]
				G		[88,126,127,128,129]
				THCA		[130]
				LSCC		[131]
				GBM		[132,133]
		THCA (C3)				[130]
		OC (C3)		TSCC		[134,135]

PCA: Prostate cancer; CRC: Colorectal cancer; HCC: Hepatocellular carcinoma; LC: Lung cancer; BC: Breast cancer; N & G: Neuroblastoma and glioma; EC: Endometrioid carcinoma; OC: Ovaric cancer; RCC: Renal cell carcinoma; P & BC: Pancreatic and biliary cancer; PC: Pancreatic cancer; GC: Gastric cancer; BT: brain tumor; PM: Pleural mesothelioma; CC: Cervical cancer; OSCC: Oral squamous cell carcinoma; TC: Thymic cancer; M: melanoma; L: Leukemia; ESCA: Esophageal cancer; G: Glioma; THCA: Thyroid carcinoma; LSCC: Laryngeal squamous cell carcinoma; GBM: Glioblastoma; TSCC: tongue squamous cell carcinoma. In parentheses: C1, CAT1; C2, CAT2; C3, CAT2.
